# Detection of colistin resistance *mcr-*1 gene in *Salmonella enterica* serovar Rissen isolated from mussels, Spain, 2012­ to 2016

**DOI:** 10.2807/1560-7917.ES.2019.24.16.1900200

**Published:** 2019-04-18

**Authors:** Antonio Lozano-Leon, Carlos Garcia-Omil, Jacobo Dalama, Rafael Rodriguez-Souto, Jaime Martinez-Urtaza, Narjol Gonzalez-Escalona

**Affiliations:** 1 ASMECRUZ Laboratory. Playa de Beluso s/n 36939, Pontevedra, Spain; 2 CI8 Research Group. Department Chemistry and Food Analysis, University of Vigo, As Lagoas-Marcosende 36310 Vigo, Pontevedra, Spain; 3 Centre for Environment, Fisheries and Aquaculture Science (Cefas), Barrack Road, Weymouth, Dorset, United Kingdom; 4 Center for Food Safety and Applied Nutrition, Food and Drug Administration, College Park, Maryland, United States

**Keywords:** Salmonella, mcr-1 gene, shellfish, mussels, whole genome sequencing, antimicrobial resistance

## Abstract

Nineteen *Salmonella* strains were isolated from 5,907 randomly selected mussel samples during a monitoring programme for the presence of *Salmonella* in shellfish in Galicia, north-west Spain (2012–16). Serovars, sequence type and antimicrobial resistance genes were determined through genome sequencing. Presence of the *mcr*-1 gene in one strain belonging to serovar Rissen and ST-469 was identified. The *mcr*-1 gene had not been isolated previously in environmental *Salmonella* isolated from mussels in Spain.

The emergence of plasmid-mediated colistin resistance due to the presence of the *mcr-*1 gene (encoding a phospho-ethanolamine transferase) was initially described in *Enterobacteriaceae* isolates in China [1] and has also been documented in Europe in human clinical cases, food products and food-animal production settings [2,3]. Increasing antimicrobial resistance in non-typhoid *Salmonella* species is considered a public health concern of the 21st century [4]. The marine ecosystem has been documented as a reservoir of antimicrobial resistant (AMR) strain and potential contributor to the globalisation of antimicrobial resistance [5].

Currently, we continue monitoring for *Salmonella* weekly in the production areas and after purification process, but until now it has not been possible to carry out whole genome sequencing on the isolated strains collected from live mussels from Galicia (north-west Spain) between 2012–16. Here, we report the findings from the sequencing and the presence of the *mcr*-1 gene and other antimicrobial resistance genes (*aac(6')-Iaa,aadA1,aadA2, blaTEM-1B, cmlA1, sul1, sul3, tet(A),* and *dfrA1)* in a strain of *Salmonella enterica* Serovar Rissen sequence type (ST) 469 that has not be found before. This is of concern, when factoring in that AMR analysis is not performed and Galicia is the third largest producer of mussel aquaculture worldwide and is considered the main supplier of mussels to the European market.

## Salmonella isolation from mussels

Between 2012 and 2016, 5,907 randomly mussel samples were taken from 15 production areas and four processing facilities in Galicia in north-west Spain. Of these samples, 19 *Salmonella* strains were isolated. Raw mussel samples (n = 5,560) were collected from one raft in each production area and 347 cooked mussel samples were collected from processing facilities. The raw and cooked mussel samples were placed in sterile bags with frozen gel-packs and transported immediately to the ASMECRUZ Laboratory (Pontevedra, Spain) in 4 °C refrigerated trucks. All samples were analysed within 12 hours of arrival at the laboratory. Mussels were removed from the sterile bags, washed with fresh water, opened under aseptic conditions and collected in a sterile bucket. Dead or broken mussels were discarded. Cooked mussels from processing facilities were analysed following the same procedure as the raw mussels.

Twenty-five grams obtained from a minimum of 15 individuals (cooked, meat and liquor for raw mussel) were weighed and 225 mL of Buffered Peptone Water (BPW) (BioMérieux/Marcy-l’Etoile, France) were added. Enrichment broths were homogenised in a stomacher at high speed for 90s and incubated at 37 °C for 24 hours. After enrichment, 750 µL were taken for DNA extraction and PCR analysis. Positive samples were streaked onto xylose lysine deoxy-cholate agar (M1031 BioMérieux /Marcy-l’Etoile France) and ChromAgar ID Salmonella (43621 BioMérieux/Marcy-l’Etoile, France). Suspected colony-forming units (CFU) were selected based on typical colonial morphology and re-isolated onto the agars mentioned above.

The strains were screened for virulence (*invA*) and antimicrobial resistance genes using conventional PCR with specific primers [6-10]. The antimicrobial susceptibility tests were performed by the VITEK 2 microbial ID/AST testing system (BioMerieux, Macy-l’ Étoile, France).

All strains were confirmed as *Salmonella* spp. by a positive PCR result for the *invA* gene (284 pb) [6]. Of the 19 *Salmonella* strains analysed, four were isolated from cooked mussels (Table 1). The antimicrobial susceptibility-resistance testing showed positive results for antimicrobial resistance (AMR) to at least four of the antibiotics tested. Two strains (AMC 90 isolated from Ría de Arosa in 2014 and AMC 270 isolated from Ría de Vigo in 2015) were resistant to eight and nine antimicrobials, respectively (Table 2).

**Table 1:** Characteristics of *Salmonella* strains from mussels sequenced and analysed, Spain, 2012–2016 (n = 19)

Strain	CFSAN number	Year/Region	Mussel source	ST^a^	Serotype^a^
AMC 28	CFSAN080361	2012/Ría Arosa	Raw	316	Montevideo
AMC 90	CFSAN080362	2014/Ría Arosa	Raw	469	Rissen
AMC 200	CFSAN080365	2014/Ría Arosa	Raw	2031	Wentworth
AMC 238	CFSAN080366	2015/Ría Arosa	Raw	34	Typhimurium
AMC 239	CFSAN080367	2015/Ría Arosa	Raw	469	Rissen
AMC 240	CFSAN080368	2015/Ría Arosa	Raw	469	Rissen
AMC 253	CFSAN080388	2015/Ría Vigo	Raw	new	Liverpool
AMC 257	CFSAN080370	2015/Ría Arosa	Cooked	4873	Offa
AMC 265	CFSAN080371	2015/Ría Arosa	Cooked	316	Montevideo
AMC 266	CFSAN080372	2015/Ría Arosa	Cooked	14	Senftenberg
AMC 267	CFSAN080373	2015/Ría Arosa	Cooked	14	Senftenberg
AMC 268	CFSAN080374	2015/Ría Vigo	Raw	19	Typhimurium
AMC 270	CFSAN080375	2015/Ría Vigo	Raw	13	Agona
AMC 289	CFSAN080379	2015/Ría Arosa	Raw	14	Senftenberg
AMC 291	CFSAN080381	2015/Ría Arosa	Raw	19	Typhimurium
AMC 294	CFSAN080382	2015/Ría Arosa	Raw	new	Typhimurium
AMC 299	CFSAN080383	2015/Ría Arosa	Raw	19	Typhimurium
AMC 301	CFSAN080385	2015/Ría Arosa	Raw	306	Bredeney
AMC 327	CFSAN080387	2016/Ría Vigo	Raw	1959	Liverpool

CFSAN: Center for Food Safety and Applied Nutrition; ST: sequence type.

^a^ The serotypes and genotype characteristics of *Salmonell*a isolates were determined by in silico analyses of the whole genome sequencing using Seqsero [11] and the multilocus sequence typing website (Enterobase).

**Table 2:** Antimicrobial susceptibility of *Salmonella* strains isolated from mussels and their antimicrobial resistance gene profiles, Galicia, Spain 2012–16 (n=19)

Strain	CFSAN number	Antibiotic resistance phenotype^a^	AMR genes^b^
AMC 28	CFSAN080361	CEF, CXM, FOX, GMN, TMN	*aac(6')-Iaa, fosA7*
AMC 90	CFSAN080362	AMP, CEF, CXM, FOX, GMN, TMN, SXT	*aac(6')-Iaa,aadA1,aadA2, blaTEM-1B,* ***mcr-1***, *cmlA1, sul1,sul3, tet(A), dfrA1*
AMC 200	CFSAN080365	CEF, CXM, FOX, GMN, TMN	*aac(6')-Iaa*
AMC 238	CFSAN080366	AMP, CXM, FOX, GMN, AKN	*aac(6')-Iaa, aph(6)-Id, aph(3”)-Ib, blaTEM-1B, sul2, tet(B)*
AMC 239	CFSAN080367	AMP, CXM, FOX, GMN, AKN	*aac(6')-Iaa, aadA2, blaTEM-1B, mph(A), sul1, dfrA12*
AMC 240	CFSAN080368	AMP, CXM, FOX, GMN, AKN	*aac(6')-Iaa, aadA2, blaTEM-1B, mph(A), sul1, dfrA12*
AMC 253	CFSAN080388	CXM, FOX, GMN, AKN	*aac(6')-Iaa*
AMC 257	CFSAN080370	CEF, CXM, FOX, GMN, TMN	*aac(6')-Iaa*
AMC 265	CFSAN080371	CEF, CXM, FOX, GMN, TMN	*aac(6')-Iaa*
AMC 266	CFSAN080372	CEF, CXM, FOX, GMN, TMN	*aac(6')-Iaa*
AMC 267	CFSAN080373	CEF, CXM, FOX, GMN, TMN	*aac(6')-Iaa*
AMC 268	CFSAN080374	CEF, CXM, FOX, GMN, TMN	*aac(6')-Iaa*
AMC 270	CFSAN080375	AMP, CEF, CXM, FOX, GMN, TMN, NAL, CIP	*aac(6')-Iaa, fosA*
AMC 289	CFSAN080379	CXM, FOX, GMN, AKN	*aac(6')-Iaa*
AMC 291	CFSAN080381	CEF, CXM, FOX, GMN, TMN	*aac(6')-Iaa*
AMC 294	CFSAN080382	AMP, CXM, SXT	*aac(6')-Iaa*
AMC 299	CFSAN080383	CXM, FOX, GMN, AKN	*aac(6')-Iaa*
AMC 301	CFSAN080385	CXM, FOX, GMN, AKN	*aac(6')-Iaa*
AMC 327	CFSAN080387	CXM, FOX, GMN, AKN	*aac(6')-Iaa*

AMC: amoxicillin/ clavulanic acid; AKN: amikacin; AMP: ampicillin; AMR: antimicrobial resistance; CEF: cephalothin; CFSAN: Center for Food Safety and Applied Nutrition; CIP: ciprofloxacin; CT: cefditoren; CTL: cefotaxime; CXM: cefuroxime; CZD: ceftazidime; ETP: ertapenem; FEP: cefepime; FOS: fosfomycin; FOX: cefoxitin; FTN: nitrofurantoin; GMN: gentamycin; IPM: imipenem; NAL: nalidixic acid; PTZ: piperacillin/tazobactam; SXT: trimethoprim/sulfamethoxazole; TGC: Tigecycline; TMN: tobramycin.

^a^ Antimicrobial susceptibility was determined with the VITEK 2 system (BioMérieux, Marcy-l'Étoile, France).

^b^ AMR genes were identified by in silico analyses of their genome sequences and queried against the AMR database (ResFinder v 3.1) hosted at the Center for Genomic Epidemiology, Technical University of Denmark (https://cge.cbs.dtu.dk/services/ResFinder).

## Characterisation of *Salmonella* strains

Whole genome sequencing and sequence processing were performed with genomic DNA extraction of 19 strains from overnight cultures using the DNeasy Blood and Tissue Kit (QIAGEN, Hilden, Germany). We sequenced the genomes using MiSeq (Illumina, San Diego, California (CA), United States (US)) using a two by 250 pair-end protocol with a V2 cartridge (500 cycles) for a minimum coverage of 40 X per genome. The DNA sequencing libraries were prepared with the Nextera XT DNA Sample preparation-kit (Illumina) and the de novo assembly for each strain was performed with CLC Genomics Workbench version 9.5.2 (QIAGEN, Valencia, CA, US). The in silico ST and serotype for those 19 *Salmonella* strains are shown in Table 1.

We identified 12 different STs among the 19 *Salmonella* strains isolated from mussels by in silico multilocus sequence typing (Table 1). Among the most prevalent STs were ST-14, 19 and 469 that corresponded to serotypes Senftenberg, Typhimurium and Rissen, respectively by in silico serotyping using SeqSero [11]. The analysis of the 19 *Salmonella* genomes showed a high diversity of AMR gene profiles (Table 2). Strain AMC 90 (CFSAN080362) identified as *Salmonella enterica* Serovar Rissen ST469 showed a high resistance in the antimicrobial susceptibility testing: (ampicillin (minimum inhibitory concentration (MIC) ≥ 32 mg/L), gentamicin (MIC < 1 mg/L), tobramycin (MIC < 1mg/L), trimethoprim-sulfamethoxazole (MIC > 320 mg/L), cefoxitin (MIC < 4 mg/L), cefuroxime (MIC = 4 mg/L), and cephalothin (MIC = 8 mg/L). The AMR genes profile was different from other isolates; *aac(6')-Iaa, aadA1, aadA2, blaTEM-1B,* *mcr-1*, *cmlA1, sul1, sul3, tet(A), dfrA1*, while other two Risen strains (AMC 239 and 240) had the same phenotypic and genotypic profile *(aac(6')-Iaa, aadA2, blaTEM-1B, mph(A), sul1, dfrA12)*. Interestingly, we discovered that strain AMC 90 carried the *mcr*-1 gene which provides polymixin E (colistin) resistance.

## Discussion and conclusions

Here, we describe the characterisation of *Salmonella* strains isolated from mussels from 2012 to 2016 in north-west Spain. The *mcr*-1 gene was detected in one of 19 Galician *Salmonella* isolates recovered from mussels. The *Salmonella* strains isolated during a previous study from molluscs from the same marine environment belonged to different serotypes and AMR profiles [12]. Marine environments may represent a source of AMR genes as they are subjected to contamination with terrestrial effluents such as agricultural wastes, discharges from human dwellings/hospitals/industry and sewage treatment plants [5]; tourism can also be a source of AMR genes, especially in the summer months when the population increases in coastal areas.

Wastewater treatment plants have been recognised as a source of AMR bacteria. Antibiotics and their metabolites access sewage through direct disposal of unused medicines or human and/or animal excretion. These compounds and bacteria are not eliminated during the treatment process. Thus, the antimicrobial agents and resistant bacteria are released into water ecosystems together with the final effluent [13].

In 2015, a study of AMR and molecular typing of *Salmonella enterica* serovar Rissen from various sources in Spain [14], showed that serovar Rissen was the second most common *Salmonella* serovar in pigs, with resistance to one or more antimicrobials being found in 78.6% of the strains and multidrug resistance in 19%. However, none of the strains came from marine or freshwater environments and none of them carried the *mcr*-1 gene. According to Cabello F et al. [5], the plasmid-associated colistin resistance mediated by the *mcr*-1 gene might have originated in aquaculture environments, with this gene already spread widely among animals and humans in China and in Europe. The current global distribution has been achieved through multiple translocations. A likely driver for the global spread is trade, in particular food animals and meat, although direct global movement by colonised or infected humans is also likely to have played a role in the current distribution. The origin of mcr-1 prior to its geographical spread remains elusive [15].

The emergence of transferable colistin resistance by *mcr*-1 undermines the revival of colistin as the ‘antibiotic of last resort’ for carbapenem-resistantce bacterial infections. Spread and global prevalence of *mcr*-1 raises a serious challenge to agricultural production and public health worldwide. Currently, the use of colistin for treatment in both animals and aquiculture environs is legal in Europe. The European Medicines Agency has raised concerns regarding the increased risk to humans from the use of colistin in animals, including aquaculture farms [16]. The One Health concept recognises the health of humans is connected to the health of animals and the environment and understanding the relative importance of the contribution of each component is important in tackling AMR [15].

To our knowledge, there is no previous identification of the presence of this *mcr-1* gene in strains of *Salmonella enterica* serovar Rissen/ST469 isolated from ready-to-eat mussels in the European marine environment or in Spain. The *mcr-1* gene is still the predominant determinant of transmissible colistin resistance, 11 more genetic variants of *mcr-1* (designated *mcr-1.2*, *mcr-1.3*…. *mcr-1.12*) were detected in different countries [17-19]. This may suggest the possibility of ongoing evolution of *mcr-*1 under some unknown selective pressure in the environment [17-19].

Our results showed the presence of AMR genes in *Salmonella* isolated from raw mussels and highlights the need for continuing surveillance of this food commodity. There is a need for public health authorities and mussel producers to ensure correct management, an efficient purification process and extensive sanitary control in ready-to-eat molluscs. The presence of a *Salmonella* strain carrying the *mcr*-1 gene in Galicia marine environment constitutes a potential risk to food safety and public health since this gene is usually located in plasmids that can easily be transferred among bacteria in this environment. Implementation of routine pathogens investigations and screening of the presence of resistance genes could contribute to a better understanding of the role of the marine environment and seafood in the transmission of AMR among human pathogens and resident bacteria.

## References

[1] Liu YY Wang Y Walsh TR Yi LX Zhang R Spencer J Emergence of plasmid-mediated colistin resistance mechanism MCR-1 in animals and human beings in China: a microbiological and molecular biological study. Lancet Infect Dis. 2016;16(2):161-8. 10.1016/S1473-3099(15)00424-7 26603172

[2] Skov RL Monnet DL Plasmid-mediated colistin resistance (mcr-1 gene): three months later, the story unfolds. Euro Surveill. 2016;21(9):30155. 10.2807/1560-7917.ES.2016.21.9.30155 26967914

[3] Campos J Cristino L Peixe L Antunes P mcr-1 in multidrug-resistant and cooper-tolerant clinically relevant Salmonella 1,4[5],12:i:- and S. Rissen clones in Portugal, 2011 to 2015. Euro Surveill. 2016;21(26):30270. 10.2807/1560-7917.ES.2016.21.26.30270 27387036

[4] de Toro M Sáenz Y Cercenado E Rojo-Bezares B García-Campello M Undabeitia E Genetic characterization of the mechanisms of resistance to amoxicillin/clavulanate and third-generation cephalosporins in Salmonella enterica from three Spanish hospitals. Int Microbiol. 2011;14(3):173-81. 22101415 10.2436/20.1501.01.146

[5] Cabello FC Godfrey HP Buschmann AH Dölz HJ Aquaculture as yet another environmental gateway to the development and globalisation of antimicrobial resistance. Lancet Infect Dis. 2016;16(7):e127-33. 10.1016/S1473-3099(16)00100-6 27083976

[6] Rahn K De Grandis SA Clarke RC McEwen SA Galán JE Ginocchio C Amplification of an invA gene sequence of Salmonella typhimurium by polymerase chain reaction as a specific method of detection of Salmonella. Mol Cell Probes. 1992;6(4):271-9. 10.1016/0890-8508(92)90002-F 1528198

[7] Swamy SC Barnhart HM Lee MD Dreesen DW Virulence determinants invA and spvC in salmonellae isolated from poultry products, wastewater, and human sources. Appl Environ Microbiol. 1996;62(10):3768-71. 8837432 10.1128/aem.62.10.3768-3771.1996 PMC168184

[8] Murugkar HV Rahman H Dutta PK Distribution of virulence genes in Salmonella serovars isolated from man & animals. Indian J Med Res. 2003;117:66-70. 12931840

[9] Cardona-Castro N Restrepo-Pineda E Correa-Ochoa M Detection of hilA gene sequences in serovars of Salmonella enterica subspecies enterica. Mem Inst Oswaldo Cruz. 2002;97(8):1153-6. 10.1590/S0074-02762002000800016 12563483

[10] Rahman H Prevalence & phenotypic expression of sopB gene among clinical isolates of Salmonella enterica. Indian J Med Res. 2006;123(1):83-8. 16567873

[11] Zhang S Yin Y Jones MB Zhang Z Deatherage Kaiser BL Dinsmore BA Salmonella serotype determination utilizing high-throughput genome sequencing data. J Clin Microbiol. 2015;53(5):1685-92. 10.1128/JCM.00323-15 25762776 PMC4400759

[12] Martinez-Urtaza J Saco M Hernandez-Cordova G Lozano A Garcia-Martin O Espinosa J Identification of Salmonella serovars isolated from live molluscan shellfish and their significance in the marine environment. J Food Prot. 2003;66(2):226-32. 10.4315/0362-028X-66.2.226 12597481

[13] Bondarczuk K Piotrowska-Seget Z Microbial diversity and antibiotic resistance in a final effluent-receiving lake. Sci Total Environ. 2019;650(Pt 2):2951-61. 10.1016/j.scitotenv.2018.10.050 30373071

[14] García-Fierro R Montero I Bances M González-Hevia MA Rodicio MR Antimicrobial drug resistance and molecular typing of Salmonella enterica Serovar Rissen from different sources. Microb Drug Resist. 2016;22(3):211-7. 10.1089/mdr.2015.0161 26295933

[15] Wang R van Dorp L Shaw LP Bradley P Wang Q Wang X The global distribution and spread of the mobilized colistin resistance gene mcr-1. Nat Commun. 2018;9(1):1179. 10.1038/s41467-018-03205-z 29563494 PMC5862964

[16] The European Medicines Agency (EMA). Updated advice on the use of colistin products in animals within the European Union: development of resistance and possible impact on human and animal health. EMA/231573/2016. London: EMA; 2016. Available from: http.www.ema.europa.eu/docs/en_GB/document_library/Scientific_guidelne/2016/05/WC500207233.pdf

[17] Yin W Li H Shen Y Liu Z Wang S Shen Z Novel plasmid –mediated colistin resistance mcr-3 in Escherichia coli. MBio. 2017;8(3):e00543-17. 10.1128/mBio.00543-17 28655818 PMC5487729

[18] Carattoli A Villa L Feudi C Curcio L Orsini S Luppi A Novel plasmid-mediated colistin resistance mcr-4 gene in Salmonella and Escherichia coli, Italy 2013, Spain and Belgium, 2015 to 2016. Euro Surveill. 2017;22(31):30589. 10.2807/1560-7917.ES.2017.22.31.30589 28797329 PMC5553062

[19] Borowiak M Fischer J Hammerl JA Hendriksen RS Szabo I Malorny B Identification of a novel transposon-associated phosphoethanolamine transferase gene, mcr-5, conferring colistin resistance in d-tartrate fermenting Salmonella enterica subsp. enterica serovar Paratyphi B. J Antimicrob Chemother. 2017;72(12):3317-24. 10.1093/jac/dkx327 28962028

